# Exercise, Diet, and Brain Health: From the Perspective of Gut Microbiota Regulation

**DOI:** 10.3390/nu17101686

**Published:** 2025-05-15

**Authors:** Li Zhang, Renhe Liu, Zheyi Song, Xin Zhang

**Affiliations:** 1 Department of Physical Education, China University of Mining and Technology, Beijing 100083, China; zhangli304036@126.com (L.Z.); 18601200091@163.com (R.L.); 2 Department of Food Science and Engineering, Ningbo University, Ningbo 315211, China; szy719792213@163.com

**Keywords:** exercise, gut microbiota, gut–brain axis, brain, diet

## Abstract

The existing body of evidence has highlighted gut microbiota as a versatile regulator of body wellness affecting not only multiple physiological metabolisms but also the function of remote organs. Emerging studies revealed a reciprocal relationship between physical exercise and intestinal microbiota, suggesting that physical exercise could enhance gut health, including regulating intestinal barrier integrity, increasing microbial diversity, and promoting beneficial microbial metabolism. Furthermore, the beneficial outcomes of exercise on the intestine may also promote brain health through the gut–brain axis. Diet is an important factor in boosting exercise performance and also greatly impacts the structure of gut microbiota. Abundant research has reported that diet alongside exercise could exert beneficial effects on metabolism, immune regulation, and the neuropsychiatric system. In this paper, we used a narrative review, primarily searching PubMed, Web of Science, and Elsevier, to review the existing research on how moderate-intensity exercise promotes gut health, and we introduced the effects of exercise on the nervous system through the gut–brain axis. We also proposed dietary strategies targeting the regulation of gut microbiota to provide guidelines for boosting brain health. This review highlights that moderate exercise and a healthy diet promote gut and brain health.

## 1. Introduction

According to an expanding body of studies, the gut microbiota is responsible for a wide range of physiological processes in the human body. In addition to being in charge of digestion and nutrient absorption, the gut microbiota is an intricate microbial ecosystem that is also vital to immunity, metabolism, and emotion [1]. The concept of the gut–brain axis further highlights the interaction between gut microbiota and the nervous system, suggesting that gut microecology directly affects brain function, including cognitive ability, emotional state, and mental health [2]. The imbalance of the gut microbiome might play a role in pathological processes associated with psychiatric and neurological diseases [3]. A stable and varied gut microbiota is ideal for immunomodulation, preserving the integrity of the gut barrier, generating metabolites that power metabolic activities, and preserving health, all of which contribute to an ideal intestinal environment [4]. Therefore, regulating the gut microbiota brings multiple benefits to the body.

Experimental studies have revealed that diet, medication use, and lifestyle have a significant impact on gut microbiota [5]. Interestingly, exercise as a healthy lifestyle has been shown to significantly improve gut health and modulate gut microbiota [6]. Data from several studies suggest that regular physical exercise can promote the growth of beneficial bacteria in the gut, thereby improving the diversity and stability of the gut microbiome [7,8,9]. Specifically, cardiorespiratory fitness training increased the relative abundance of *Clostridiales*, *Roseburia*, *Lachnospiraceae*, and *Erysipelotrichaceae*, which are the groups that produce butyrate [10]. Moreover, beneficial species including *Roseburia hominis*, *Akkermansia muciniphila*, and *Faecalibacterium prausnitzii* were increased in women with exercise [11]. Other than regulating the microbiota abundance, moderate exercise can also help to enhance intestinal barrier integrity and attenuate inflammatory response [12]. Exercise increases beneficial bacteria and promotes short-chain fatty acid (SCFA) production through the modulation of the gut microbiota [8]. SCFAs mediate intestinal barrier protection via three key pathways: (1) enhancing intestinal epithelial cell differentiation, (2) upregulating tight junction proteins, and (3) stimulating anti-inflammatory factor release, thus preserving mucosal integrity [13].

There are compelling indications that aerobic exercise may contribute to boosting cognitive functions, which has been demonstrated in exercise-associated increases in gray matter volumes across various brain regions. Furthermore, longitudinal research suggests that regular aerobic activity may exert potential neuroprotective effects, involving enhanced neurogenesis, improved cerebral blood flow, and reduced neuroinflammation [14]. For example, exercise may help Alzheimer’s disease (AD) patients’ cognitive performance or slow down cognitive decline [15]. However, recent research has suggested that gut microbiota may be the critical mediator of exercise’s cognitive-enhancing benefits. Kang et al. used running wheels as a forced exercise paradigm for 16 weeks in adult mice, showing improvement in memory and a marked shift in the gut microbial community. The authors hypothesized that the observed correlation between specific gut microbial taxa abundance and contextual memory performance may serve as possible microbiome indicators of exercise’s cognitive effects [16]. Recent studies have shown that when mice on a high-fat, high-cholesterol (HFHC) diet received feces from voluntarily exercising mice, their learning and memory abilities improved compared with the sedentary group. The outcomes of cognitive improvement may be related to the increased abundance of beneficial bacteria and elevated SCFAs levels, consequently improving synaptic plasticity-related proteins and neuroinflammation [17].

Modern socioeconomic and cultural changes have gradually changed our lifestyles and habits, including changes in dietary patterns, like eating more prepared foods, processed foods, and foods rich in additives. These changes in dietary patterns alter the gut microbiota, increasing the risk of metabolic diseases and chronic diseases and impairing brain health [18]. Therefore, reasonable dietary structure and nutritional elements are key to helping the body maintain a balanced intestinal microbiota. Diets rich in dietary fiber, fermented foods, and foods with prebiotic activity can regulate the structure of gut microbiota and produce metabolites that are beneficial to organs and the brain. A healthy dietary pattern can ensure adequate nutrient intake and prevent metabolic and neurological diseases. Dietary patterns such as the Mediterranean diet (MD) are conducive to increasing the diversity of the gut microbiota, and the antioxidant components of the MD, such as polyphenols, can effectively prevent the proliferation of harmful bacteria [19]. The plant-based diet has been shown to improve the dysbiosis of Firmicutes and Bacteroidetes and enhance intestinal barrier function, and it is associated with high SCFA levels [20]. In addition, the Mediterranean–DASH intervention for neurodegenerative delay (MIND) diet protects brain health and helps reduce the risk of AD and dementia [21].

In this article, we first outline the advantages of moderate exercise for intestinal health. Next, in light of the current findings, we address how exercise affects brain health and highlight the mediation function of gut bacteria in this process. Taking into account that the gut microbiota may mediate the positive benefits of exercise on the brain, finally, we discuss the advantages of healthy dietary patterns from the perspective of intestinal microbiota regulation, including the MD, the MIND diet, dietary approaches to stop hypertension (DASH), and a plant-based diet. This review aims to (1) demonstrate recent evidence of the positive effects of moderate-intensity exercise training on gut health, (2) reveal the mechanism of action of exercise on brain health through the gut–brain axis, and (3) provide dietary recommendations for promoting gut and brain health. It is hoped that this review may provide new insights into how exercise promotes brain function and provide guidance on dietary patterns that promote gut health.

## 2. Study Design

### 2.1. Databases

We primarily searched PubMed, Web of Science, and Elsevier, and also included manual searches of key journal lists.

### 2.2. Selection Criteria

We focused on studies addressing “exercise and gut”, “exercise and brain health”, “diet and gut”, “gut and brain”, and “diet and brain”, prioritizing recently published journals and important research.

### 2.3. Analysis Approach

As a narrative review, our analysis aims to synthesize a wide range of topics rather than statistical summary data.

## 3. Moderate Exercise Is a Regulator of Gut Health

Moderate exercise (around 30 min of moderate-intensity activity five to seven times a week) can provide multiple health benefits, although the amount of exercise varies among individuals [22]. Indeed, endurance athletes are highly susceptible to gastrointestinal problems like gastrointestinal bleeding, diarrhea, and nausea, and intense physical activity may trigger gut inflammation and undermine intestinal epithelial tight junction proteins [23]. Therefore, here, we mainly discuss the beneficial impacts of moderate exercise intensity on the gut.

### 3.1. Exercise Regulates Intestinal Barrier Integrity

The length and intensity of exercise have an impact on intestinal permeability. Exhaustive exercise was observed to cause intestinal barrier disruption, gut inflammation, and increased intestinal permeability in animal models [24]. Male runners with high-intensity training showed increased gastrointestinal damage and permeability indicators [25], which increase the risk of a “leaky” gut. It is typified by high dysfunction of the intestinal epithelial barrier and excessive permeability, which let toxic substances and dangerous microbes pass into the bloodstream and have negative impacts on various organs and systems [26]. However, enhanced intestinal barrier integrity has been observed following low/moderate-intensity exercise in limited studies. When mice underwent 30 min daily swimming for seven days, stress-related barrier dysfunction was alleviated, along with reduced bacterial translocation to mesenteric lymph nodes. This antimicrobial improvement was linked to elevated antimicrobial peptide production [27]. Allen et al. discovered that germ-free (GF) mice receiving bacteria from mice with running wheel-attenuated clinical responses to colitis feature an alleviated colon mucus layer, mitigated colonic immune cell infiltration, and augmented anti-inflammatory gene expression [28].

The impact of exercise on the intestinal barrier in people with metabolic diseases is being investigated further. Moderate treadmill activity significantly restored the decrease in the variety and relative abundance of gut microbiota caused by a high-fat diet (HFD). Additionally, exercise mice had higher levels of colonic ZO-1 and occludin protein expression [29]. It is thought that the stress-inducible protein Sestrin2 helps epithelial cells survive and recover. Mice exercising on a treadmill for 5 days a week for 15 weeks significantly reversed HFD-induced intestinal barrier dysfunction. The author speculated that Sestrin2 takes part in regulating intestinal permeability [30]. Human clinical studies have demonstrated exercise’s ability to improve intestinal barrier integrity, corroborating findings from rodent models. Notably, Pasini et al. investigated the exercise effects on the composition of gut microbiota and intestinal permeability in type 2 diabetes (T2D) patients. Following a 5-month regimen combining endurance, resistance, and flexibility training, participants showed reduced gut dysbiosis, decreased intestinal permeability, and lower systemic inflammation markers [31]. Similarly, in people with insulin resistance, moderate aerobic training for 2 weeks reduced systemic and intestinal inflammatory markers of lipopolysaccharide (LPS) binding protein and TNF-α [32]. These results may be the result of exercise improving the integrity of the intestinal barrier.

### 3.2. Exercise Regulates Gut Microbiota Composition

At present, the results of exercise on promoting gut microbiota diversity are controversial. According to one study, short-term endurance exercise fails to induce significant gut microbiota changes in older adults, as evidenced by the fact that there were no differences in α-diversity index changes comparing exercise and control periods [33]. Similarly, Bressa et al. observed that inactive periods and breaks were associated with microbiota, but exercise interventions showed no meaningful alterations to microbial diversity or richness of microbiota in both active and sedentary women [11]. These findings align with Rettedal et al.’s demonstration that short-term high-intensity interval training did not impact the overall bacterial diversity or community structure [34]. Allen et al. presented a study showing that 6 weeks of aerobic exercise training altered the gut microbiota composition in previously sedentary lean adults and adults with obesity. Exercise-induced shifts in SCFA-producing taxa (*Faecalibacterium* spp. and *Lachnospira* spp.) and genetic machinery (BCoAT) were more substantial in lean subjects than in participants with obesity [35]. The differences in these results may be due to gender, age, obesity, and duration of exercise, and the sample size is also an important factor. In addition, dietary differences were also important variables in the study. Professional rugby players had higher gut microbiota richness than BIM-matched and non-matched populations; however, this difference was mostly caused by dietary differences [36]. Moreover, mice that voluntarily run on wheels only exhibit increased species richness when fed high-fat diets, not low-fat diets, highlighting the significance of diet in researching how exercise affects gut microbiota [37].

Exercise has been demonstrated in numerous studies to change the gut microbiota’s composition and increase the enrichment of health-related bacteria. According to a meta-analysis, exercise dramatically alters the Firmicute/Bacteroidete ratio in adults, with greater changes in diversity indexes observed in females and older adults [6]. A non-randomized comparative trial study examined exercise effects on gut microbiota composition in elderly women. Results showed that aerobic exercise training significantly elevated *Bacteroides* [38]. Compared with sedentary women, active participants revealed higher levels of beneficial microbes (*Faecalibacterium prausnitzii*, *Roseburia hominis*, and *Akkermansia muciniphila*) [11]. Six-week endurance exercise training greatly modified microbial composition and functions, particularly increasing *Akkermansia* while reducing *Proteobacteria* [39]. For subjects with metabolic diseases, the effect of exercise on intestinal microbiota may be more significant. Exercise significantly reduced the *Proteobacteria* phylum and *Gammaproteobacteria* class and raised *Blautia*, *Dialister*, and *Roseburia* genera in children with obesity, which had a profile similar to healthy children [40]. In addition, aerobic exercise significantly increased *Bifidobacterium* in women with excessive weight, but *Lactobacillus* showed no significant change in response to intervention [41].

### 3.3. Exercise Promotes Beneficial Intestinal Metabolism

Exercise-induced gut microbiota modulation results in commensurate metabolic alterations. Higher physical activity levels and improved cardiorespiratory fitness correlate with increased amounts of SCFAs and fecal bacteria α-diversity [42]. A previous study demonstrated that endurance exercise upregulates key SCFA-producing pathways, including butyrate synthesis via the BCoAT gene and propionate production through mmDA gene modulation, thereby enhancing the gut microbiome’s SCFA-generating capacity [35]. Interestingly, Huang et al. found that the gut microbiota remodeling feature caused by hypoxia boosted *Akkermansia* and *Bacteroidetes* genera proliferation and increased the production of SCFAs. In addition, SCFA concentrations were correlated with promoted mitochondrial synthesis and ameliorated exercise fatigue biochemical parameters [43]. Importantly, a recent study by Magzal et al. reported that a less active group of older persons with insomnia had higher levels of SCFAs. The more active group had significantly greater levels of the microbial families Erysipelotrichaceae and Peptococcaceae, as well as the genus *Peptococcus* [44]. Further, exercise can also mediate bile acid metabolism through gut microbiota. Following the administration of fecal samples from exercise-trained mice, GF animals demonstrated increased skeletal muscle levels of AMP-activated protein kinase (AMPK) and insulin growth factor-1 and promoted plasma bile acid deconjugation, demonstrating how the gut microbiota alters circulating bile acids to mediate exercise-induced metabolic benefits in mice [45]. Moreover, HFD-fed rats after 60 min of combined aerobic-resistance training effectively showed gut microbiota remodeling that prevented gut–liver axis dysregulation and improved bile acid homeostasis [46]. In addition, exercise-induced alterations in gut microbial communities were linked to certain metabolic pathways, including increased levels of *Methanobrevibacter smithii*, a methane-producing archaeon linked to enhanced energy and carbohydrate metabolism in professional cyclists. Furthermore, elevated *Prevotella* levels were correlated with changes in amino acid and carbohydrate metabolic pathways [47]. In addition, the genus *Veillonella* was increased in marathon runners, which is a bacterium that utilizes lactate as the only carbon source [48].

## 4. Effects of Exercise on Brain Health Through the Gut–Brain Axis

Many studies have suggested that moderate exercise can promote healthy brain function, including enhancing memory, improving mood, and improving cognitive performance. One study provides direct evidence that exercise alters gut microbiota profiles and plays a role in cognitive disorders [17]. Indeed, exercise plays a key role in a variety of neurological diseases and can promote the reduction of oxidative stress and release of anti-inflammatory factors, regulate microglia, promote the production of neurotransmitters, etc. However, whether these beneficial effects are regulated by microbiota is unclear. Thus, more studies are needed to confirm whether exercise-induced changes in gut microbiota mediate brain health. We next explore the health benefits of exercise on brain health and highlight the mediating role of gut microbiota in this process in conjunction with the existing research.

### 4.1. Relieves Neuroinflammation

Regular exercise can help minimize the risk of chronic diseases thanks to its anti-inflammatory properties. Potential mechanisms involve decreased Toll-like receptor (TLR) expression on inflammatory cells, enhanced secretion of anti-inflammatory cytokines from working skeletal muscle, and decreased visceral fat mass [49]. Recently, the vagus nerve has also attracted increasing attention in the gut–brain communication during exercise. Exercise training can regulate autonomic nervous system responses and affect gut–brain communication through the vagus nervous system. Moderate aerobic exercise helps to enhance heart and lung function and promote vagal activity and sympathetic vagal balance [50]. The vagus nerve plays an anti-inflammatory role by regulating the release of neurotransmitter acetylcholine from T cells, binding to receptors on the surface of immune cells such as macrophages, inhibiting the NF-κB signaling pathway, and reducing the release of proinflammatory cytokines [51]. Studies have shown that vagus nerve stimulation reduces the inflammatory response to toxins in the body. However, vagotomy without electrical stimulation increased TNF-α levels [52]. In addition, muscle contraction during exercise releases IL-6, which signals to the brain through the vagus nerve, promoting the systemic release of anti-inflammatory mediators, including IL-1ra and IL-10, while simultaneously suppressing proinflammatory cytokine TNF-α generation [52]. It is interesting to note that exercise can alter inflammation in the central nervous system (CNS) in addition to its effects on the peripheral nervous system [53]. Microglia are resident immune cells of the CNS and have a role in maintaining central system homeostasis and immune defense [54]. They are classified into two categories: proinflammatory (M1) and anti-inflammatory (M2). Activated microglia increase the release of inflammatory factors and reactive oxygen species (ROS) and can also release brain-derived neurotrophic factor (BDNF) and neurotrophins to help in nerve recovery [55]. However, upon encountering injury or disease, microglia are adversely activated, resulting in a chronic inflammatory environment [56]. Physical exercise has been demonstrated to alter microglia activity and lower inflammation in the brain. Enhancing physical exercise has been shown in animal studies to lower microglial activation while simultaneously enhancing synaptogenesis and neurogenesis [57]. According to Kim et al., treadmill running decreased Aβ plaque burden, neuroinflammation, and microglia death, as well as attenuated AD progression and cognitive decline [58]. Another research study revealed that wheel running elevated the percentage of microglia positive for insulin-like growth factor (IGF-1), indicating that exercise promotes a neuroprotective microglia phenotype [59]. Casaletto et al. suggested that higher physical activity levels correlate with reduced microglial morphological activation, and physical activity may improve synaptic and cognitive resilience associated with AD by attenuating pro-inflammatory microglia phenotype [60]. It has been verified that 12-week treadmill exercise can significantly inhibit neuroinflammation via modulating active microglia from the M1 to M2, which in turn increases anti-inflammatory mediators and decreases proinflammatory factor expression in the hippocampus [61].

Importantly, the microglial function is closely related to gut microbiota conditions. The changes in intestinal microbiota and LPS produced by gut microbiota can lead to neuroinflammation. LPS can cross the gut–blood barrier and the blood–brain barrier (BBB), then be bound by TLR4 on microglia to activate the immune response and promote the release of proinflammatory factors [62]. The regulating effect of physical exercise on gut microbiota ecology may be part of the reason for the improvement of neuroinflammation. Littlefield et al. reported that voluntary wheel running may prevent LPS-induced neurogenesis and enhance neuroprotective microglia phenotype in the elderly brain. Moreover, exercise significantly increased the proportion of microglia co-labeled with BDNF [63]. Recent studies evidenced that aerobic training enhances the cognitive and spatial learning abilities of mice treated with LPS via suppressing microglia-driven neuroinflammation through BDNF–FNDC5–irisin pathway modulation [64]. Preoperative exercise intervention removed metabolic syndrome risk for the aberrant surgical phenotype, avoided cognitive decline and neuroinflammation, and improved gut microbiota diversity in metabolic syndrome [65]. Moreover, exercise enriched gut microbial diversity, attenuated LPS displacement, and alleviated abnormally activated microglia in AD mice. The outcomes of exercise also effectively modulate gut microbiota and the intestinal barrier, indicating the potential involvement of microbiota/gut–brain axis mechanisms in exercise’s effect on neuroinflammation [66]. Further, SCFAs can cross the BBB to influence CNS cells directly, which can maintain the normal physiological function of microglia and prevent microglia overactivation [67]. Butyrate was shown to reduce LPS-induced microglial inflammation, hence exerting an anti-inflammatory effect [68]. In addition to increasing butyrate generation and improving butyrate-producing fecal bacteria, exercise also reduces the relative abundance of phyla that produce LPS [69]. Experiments have shown that the gut microbiota of exercising mice have a higher proportion of butyrate microbes in the gut [28]. Research has shown that SCFAs might be critical for improving HFHC-induced cognitive impairment. The exercise-induced gut microbiota increased SCFA receptor expression in the proximal colon tissue and the total SCFA levels, which might account for the upregulation of BDNF and synaptic proteins [17].

### 4.2. Regulates Neurotrophic Factors and Neurotransmitters

The nervous system contains large amounts of BDNF, which is essential for neuron survival, development, differentiation, and plasticity. The effects of exercise on synaptic plasticity and cognitive function have been demonstrated to be mediated by BDNF. Voluntary aerobic exercise has been shown to consistently raise BDNF mRNA and protein in the hippocampus in animal studies [70]. An exercise-mediated improvement in spatial learning was eliminated when BDNF activity was inhibited during exercise [71]. A meta-analysis examined the effect of exercise on BDNF levels in different exercise paradigms. The results showed a significant rise in BDNF, and consistent exercise further strengthened the training’s impact on BDNF levels [72]. Similarly, healthy people’s peripheral blood BDNF levels rose after acute exercise. However, the impact of exercise on BDNF varies depending on how it lasts and differs for men and women [72,73,74]. Interestingly, intestinal microbiota has been linked to BDNF regulation. Lower levels of BDNF expression were seen in the cerebral cortex and hippocampal areas of GF animals [75]. A rodent study displayed that oral supplementation with *Bifidobacterium* increases BDNF expression, while aerobic exercise in piglets also revealed elevated *Bifidobacterium* in the gut [76]. In addition, *Lactobacillus* intervention could reverse BDNF and its receptor tyrosine kinase receptor B (TrkB) [77]. However, the effect of aerobic exercise on *Lactobacillus* and *Bifidobacterium* has not been fully studied. A pilot investigation into the impact of aerobic exercise on *Lactobacillus* and *Bifidobacterium* in overweight women revealed that there were notable time–exercise interaction effects on Bifidobacterium and time effects on *Lactobacillus*. Only *Bifidobacterium* species were adversely connected with IL-6 levels, and changes in these counts in response to exercise were negatively connected with weight [41].

The gut microbiota plays a key role in gut–brain communication by generating neuroactive molecules. The CNS’s predominant inhibitory neurotransmitter, γ-aminobutyric acid (GABA), is crucial for both brain activity and gastrointestinal physiological processes. Moderate exercise augmented hypothalamic GABA levels, leading to decreased resting blood pressure and sympathetic tone [78]. According to the experiments of Liu et al., the contents of propionate and GABA increased significantly in responders after 12 weeks of exercise intervention [79]. Gut microbiota can synthesize and regulate neurotransmitter activity and interact with the CNS to regulate brain functions. Several microbial species such as *Bacteroides* can convert glutamate into GABA, and *Lactobacillus* genus strains have been shown to be GABA products. In addition, dopamine and noradrenaline can be synthesized in the gastrointestinal tract during stress [80]. Physical exercise stimulates intestinal sensory nerves, leading to suppressed monoamine oxidase expression that normally breaks down dopamine in the ventral striatum, thereby enhancing dopaminergic synthesis and signaling. Furthermore, the reduced expression of dopamine-degrading enzymes in exercise mice was not observed in microbiota-depleted mice [81]. Moreover, probiotic consumption alleviated the reduction in circulating tryptophan during exercise, suggesting that gut microbiota can regulate neurotransmitter levels and influence serotonin (5-HT) metabolism in the brain [80].

### 4.3. Supports Positive Mood

There is an interaction between exercise and emotional stress. Exercise can ameliorate bad mood or increase stress (e.g., during competition in professional athletes), while stress can also affect exercise performance and bowel function. The activity of the hypothalamic–pituitary–adrenal axis (HPA) is closely related to mood states. Exercisers who exceed 60% of their maximal oxygen intake (VO_2_max) or engage in continuous exercise (>90 min) exhibit increased HPA axis activation and alteration of their gut microbiome [82]. The HPA axis is influenced by the gut microbiota diversity, where bacterial depletion leads to exaggerated HPA responses to psychological stress [83]. Rodents and human trials have shown that *Bifidobacterium* species can reverse HPA overactivation and alleviate anxiety and depressive symptoms. While *Bifidobacterium* is also affected by aerobic exercise, studies in mice show that voluntary wheel running increased *Lactobacillus* and *Bifidobacterium* abundance, whereas forced exercise conversely reduced *Lactobacillus* levels [84].

**Figure 1:**
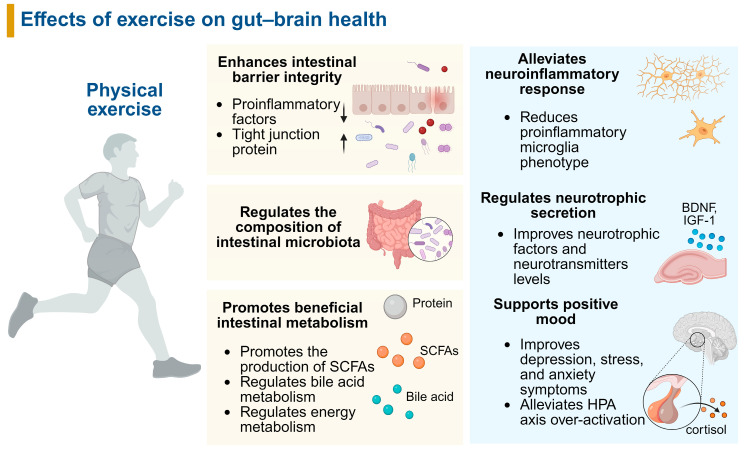
The effects of exercise on gut–brain health. SCFAs, short-chain fatty acids; BDNF, brain-derived neurotrophic factor; IGF-1, insulin-like growth factor 1; HPA axis, the hypothalamic–pituitary–adrenal axis; ↑: increase; ↓: decrease.

5-HT is a key neurotransmitter and is often known as one of the primary mood regulators. Low levels of 5-HT are associated with anxiety and depression, whereas high levels are associated with mood improvement [85]. It has been observed that gut microbiota can regulate 5-HT synthesis and act as a crucial mediator in the gut–brain axis. Enterochromaffin cells create about 95% of the body’s 5-HT, which is involved in enteric motor and sensory processes. 5-HT levels have also been demonstrated to be modulated by exercise. Low-speed running appears to raise brain 5-HT levels and reduce depressive and anxious behavior, whereas high-speed running elevated corticotrophin-releasing hormone (CRH) expression [86]. Moreover, swimming for 30 min every day for six days a week for 1 month raises 5-HTP concentrations in a rat hypothalamus and brain stem model [82]. Liu et al. explored the mechanism of exercise in improving depression and found that 8 weeks of running exercise successfully suppressed microglia activation along the gut–brain axis and enhanced the intestinal epithelial barrier. Running could increase dopamine and 5-HT expression in the hippocampus of mice, and these results may be caused by the improvement of the intestinal barrier [87]. Recent research revealed that running can modulate gut microbial diversity and tryptophan-metabolizing capabilities, inducing significant alterations in tryptophan metabolic pathways within the microbiota/gut–brain axis (including the gut, blood, hippocampus, and brainstem). *Romboutsia* and *Akkermansia muciniphila*’s symbiotic relationship shows the potential to alter the microenvironment and affect tryptophan transport to the brainstem and hippocampus [9]. These results demonstrate how gut bacteria regulate the effects of exercise on brain function (Figure 1).

## 5. Dietary Strategies Aimed at Regulating the Gut Microbiota

Adequate nutrients can not only supplement nutrition for exercise and help recovery after exercise but also help to shape a healthy intestinal microenvironment. Healthy dietary structure can form different gut microbiota and bring a variety of health benefits, such as preventing neuropsychiatric diseases. For example, plant-based diets are rich in dietary fiber and prebiotic active ingredients, which can promote body health by promoting the proliferation of beneficial bacteria and increasing beneficial metabolites [20]. We present the characteristics of the different dietary structures in Table 1. Here, we discussed the protective effects of several different healthy dietary patterns on the brain by targeting the modulation of gut microbiota.

### 5.1. MD

MD has demonstrated protective effects against various diseases, including reduced risks of heart disease, stroke, and memory loss. MD features a high dietary intake of vegetables, whole grains, legumes, and fruits, with moderate consumption of fish and olive oil as the primary sources of fats. MD also includes limited red wine consumption with meals. Some research showed the effects of MD and exercise on gut microbiota. A cross-sectional study showed significant dissimilarities in the microbiota composition on MD adherence and physical activity. Subjects with lower BMI, lower levels of physical activity, and lower MD adherence had higher *Paraprevotella* abundance [88]. One study examined how lifestyle (diet and exercise) interventions affected the gut microbiota of men and women with obesity/overweight. It was discovered that alterations in gut microbiota were brought on by both physical activity and weight loss, and changes in *Lachnospiraceae* were positively associated with MD adherence [94]. The occurrence of particular strains in the gut microbiota is linked to typical food components found in the MD. For instance, the intake of cereal is associated with *Bifidobacterium*, *Faecalibacterium*, and Coprococcus. The consumption of olive oil and red wine is associated with *Faecalibacterium* [95]. Mitsou et al. revealed that subjects with high adherence to the MD had lower *Esherichia coli* counts and an increased *Bifidobacteria*:*E. coli* ratio, as well as increased *Candida albicans* colonization patterns [96]. Preclinical and clinical research indicated that MD rich in virgin olive oil increased *Lactobacillus* and *Bifidobacterium* in the intestine and significantly decreased pro-inflammatory cytokines and oxidative stress in overweight subjects [97]. In addition, olive oil also promotes the development of intestinal immunoglobulin A (IgA), which protects the intestinal mucosa and maintains gut microbial balance [98]. As the most important dietary component of MD, olive oil is rich in hydroxytyrosol and oleuropein, which have strong antioxidant action and neuroprotective effects [99]. Accumulating evidence supports that olive oil-rich MD can significantly reduce the risk of AD. Chen et al. compared olive oil and camellia oil against cognitive impairment in rats, and the results showed that the abundance of *Alistipes*, *Odoribacter*, and *Parabacteroides* was increased and *Prevotellaceae* decreased in the olive oil group, in which the microorganisms have been found to demonstrate improvements in psychiatric disorders. In addition, it also suggested that the effects of the minor components of olive oil on the inflammatory response in the brain can be directly influenced by the action of microbiota and phenols [100].

### 5.2. DASH Diet

The DASH diet was designed to help prevent or treat high blood pressure and advocates increasing the consumption of vegetables, fruits, whole grains, and low-fat milk; eating moderate amounts of nuts and legumes; and limiting foods high in added sugars and saturated fats. The DASH diet has more dietary fiber, calcium, magnesium, and potassium plasma intake and focuses on fresh and unprocessed or less processed foods, and strict followers usually limit their sodium intake to less than 1500 mg [101]. The DASH diet is a stricter MD that is lower in fat, severely limits alcohol intake, and controls sodium intake, including from various seasonings and foods. Similar to the MD, the DASH diet can also promote the expansion of protective microbes and SCFA generation, as well as decrease inflammatory markers [102]. Recent research showed that a low-calorie DASH diet significantly lowered the Firmicute and Bactericide (F/B) ratio and significantly reduced the trimethylamine N-oxide and LPS levels [103]. In addition, DASH is negatively associated with biological aging and indicates that the *Synergistetes* phylum and its component genus *Pyramidobacter* may be potential mediators [104]. Moreover, consuming too much salt is thought to be a major risk factor for cognitive impairment, which can lead to a significant increase in plasma IL-17 depending on the growth of TH17 cells in the small intestine. This can further exacerbate cognitive impairment by preventing the phosphorylation of endothelial nitric oxide synthase and reducing the production of nitric oxide in brain endothelial cells [105]. According to Hu et al., mice given a diet high in salt showed poor performance in learning and memory and had much lower levels of Bacteroidetes and Proteobacteria, but showed increased *Lachnospiraceae* and *Ruminococcaceae*. High-salt diets reduced the SCFA concentration and induced both BBB dysfunction and microglial activation in the brain, increasing IL-1, IL-6, and TNF-α expression levels [106]. Thus, the low intake advocated by the DASH diet may maintain healthy brain function by affecting gut microbiota and related metabolism.

### 5.3. MIND Diet

**Figure 2:**
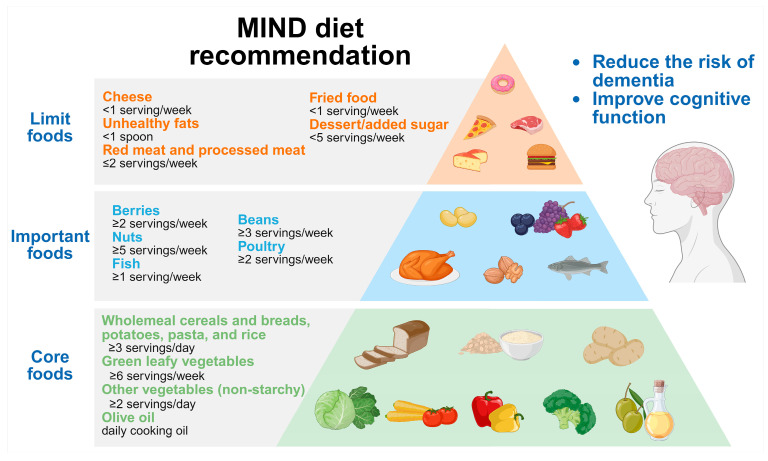
The diet structure of the MIND diet.

The MIND diet, also known as the healthy brain diet, combines the strengths of the DASH and MD diets with an emphasis on dietary components that are neuroprotective and protective against dementia. The Mediterranean and DASH diets’ nutritional components served as the foundation for the MIND diet, which differs in that it places an emphasis on leafy greens and berries without emphasizing fruits (the DASH diet recommends four to five servings per day), potatoes, dairy, and fish (more than six meals per week in the Mediterranean diet). Figure 2 shows the structure of the MIND diet. A long-term study has reported that adherence to the MIND diet significantly slows cognitive decline associated with aging [90]. Meanwhile, a prospective study showed that adherence to the MIND diet can reduce the risk of AD and influence its progression [21]. The neuroprotective effects of the MIND diet may be linked to its preference for berries over other fruits. Experiments in rodents have revealed that dietary supplementation with berries improves learning and memory and positively modulates gut microbiota. Mice fed with Nordic berries showed enhanced spatial memory and modulated inflammatory status in the brain. Interestingly, mice consuming a high-fat diet supplemented with bilberries and grape juice—rich sources of anthocyanins and proanthocyanins—exhibited a significant increase in *Akkermansia muciniphila* relative abundance compared to control groups [107]. *A. muciniphila* has been demonstrated to exert beneficial outcomes on cognitive performance by enhancing the intestinal mucosal barrier, promoting beneficial metabolites, and directly signaling outer membrane proteins [108]. In addition, anthocyanins have the ability to stimulate the growth of *Bifidobacterium* and *Lactobacillus*, two helpful gut microbes. Ellagitannin, which is present in blackberries and strawberries, serves as a substrate for bacteria in the colon, primarily those belonging to the *Clostridium* and *Bacteroides* groups. Flavonoids present in berries undergo metabolic transformation through the gut microbiota to produce more bioavailable metabolites, promote the neuroprotective metabolites, and modify the gut microbiota’s composition [109]. Green leafy vegetables are rich in dietary fiber, vitamins, and flavonoids that have been linked to a decreased risk of cognitive impairment. Additionally, some dietary nutrients feature antioxidant, anti-inflammatory, and anti-neurotoxic properties that protect the brain. Additionally, the MIND diet recommends the consumption of olive oil and a small amount of wine, which, as mentioned above, can help prevent neurodegenerative diseases and slow cognitive decline.

### 5.4. Plant-Based Diet

The plant-based diet is a dietary pattern that emphasizes plant-based foods, such as vegetables, fruits, whole grains, legumes, and nuts as the main dietary sources. This dietary pattern reasonably controls or avoids the intake of animal foods such as meat and seafood, emphasizes plant foods as the core of the diet, and pays attention to the balanced intake of nutrients, which has a variety of health benefits. Plant-based diets are gaining popularity around the world as health concerns and environmental awareness grow [92]. The fermentation of indigestible carbohydrates is significantly influenced by the gut microbiota. Plant-based diets are rich in dietary fiber intake, which promotes microbes that produce butyrate and other SCFAs, exerting anti-inflammatory effects and strengthening intestinal barrier function [20]. One strictly controlled cross-over interventional study revealed that meals consisting solely of plant or animal products modify the structure of microbial communities and change genetic expression within 5 days. Notably, *Prevotella* is one of the main sources of variation in intestinal microbiota between individuals, which is thought to be sensitive to long-term fiber ingestion but was reduced during the intake of animal-based diets by vegetarians [110]. Previous research has found that diets high in carbohydrates (plant-based diet) were correlated with more *Prevotella*. This might be linked to the increased synthesis of SCFAs and be beneficial for keeping inflammatory processes at normal levels [93]. In addition, vegans and vegetarians have a higher number of bacterial genera associated with the Bacteroidetes phylum, which has enzymes (CAZymes) that encode carbohydrate-active enzymes to degrade indigestible carbohydrates. Meanwhile, dietary fibers increase lactic acid bacteria like *Ruminococcus*, *E. rectale*, and *Roseburia*, and reduce *Clostridium* and *Enterococcus* species [111]. Furthermore, plant foods contain a variety of polysaccharides, polyphenols, and peptides with bioactive properties. In addition to their direct effects on targets, macromolecules can be used by intestinal microbes to regulate gut microbiota and generate metabolites, which regulate the CNS via the gut–brain signal. For instance, the phenolic compound epigallocatechin gallate has been reported to increase both bacterial and species richness, decrease Desulfovibrio, and raise the abundance of *A. muciniphila* and *Bifidobacterium*. The modulation of gut microorganisms is related to decreased microglia activation and neuroinflammation in the brain [112]. Sesamol-fed AD animals showed decreased neuronal damage, reduced neuroinflammatory responses, and enhanced memory and learning. Additionally, it altered the gut microbiota by observing a decrease in the relative abundance of *Bacillaceae* and *Clostridium* and an increase in the relative abundance of *Bifidobacterium* and *Rikenellaceae*, as well as significantly increasing SCFA levels in mice [113]. Soybean is rich in vital fatty acids, dietary fiber, soybean isoflavones, and high-quality protein, which is a major component of protein for plant-based diets. The main bioactive byproduct of the gut microbiota’s transformation of soy isoflavone daidzein is S-equol. S-equol, but not soy isoflavones, was linked to strengthened cognitive function and dementia, according to observational and clinical research conducted in Japan [114]. Moreover, dietary-resistant starch can play a neuroprotective role, and the cognitive improvement is attributed to the positive remodeling of gut microbial metabolism, including the increase of SCFAs and the decrease of branched-chain amino acid levels [115]. Fructo-oligosaccharides are oligosaccharides that occur naturally in a variety of plants. It has been revealed that fructooligosaccharides can alleviate depression-like behaviors, repair intestinal epithelial damage, and alleviate microbiota dysbiosis. In addition, it has been demonstrated that *Morinda officinalis* fructooligosaccharides increase the number of *Cyanobacteria* in the microbial phylum, which was known for secreting the pharmacologically significant metabolite H2S, a metabolite with antidepressant effects [116].

## 6. Conclusions and Future Perspectives

Studies have indicated a dynamic interaction between intestinal microbiota and physical exercise. Moderate exercise has the health benefits of strengthening the intestinal barrier, regulating gut microbiota, and promoting the production of beneficial gut metabolites. Gut microbiota is known to improve mood symptoms, including stress and anxiety, and may mediate positive benefits on the brain. This experiment showed dramatically increased BDNF and neurotransmitter concentrations, improvements to cognitive ability, and stabilization of mood. Healthy dietary patterns can reshape gut microbiota and boost brain health, such as polyphenols, which can reduce neuroinflammatory responses by regulating gut microbiota. However, few studies have explored the effects of exercise on brain function from the perspective of gut microbiota. Most studies have only observed the correlation between exercise and changes in intestinal microbiota, as well as between exercise and the improvement of brain function. However, there is a lack of direct evidence that intestinal changes are an important way for exercise to affect the brain. In addition, the metabolic pathways involved in exercise are complex, such as SCFAs, bile acid, tryptophan metabolism, and other molecules, and their effects on the nervous system are not clear. Future studies should be based on 16S rRNA sequencing and also require longitudinal combined analysis of metagenomic, metabolomic, and brain imaging techniques. In addition, mouse models cannot fully simulate the diversity of human exercise (such as exercise type and psychological motivation), and human trials have small sample sizes. Therefore, large-scale cohort studies combining metagenomic and brain health information to develop precise exercise intervention strategies for different populations (such as those with obesity, metabolic problems, and AD) are the focus of future research. The GF animal models or fecal microbiota transplantation can be used to clarify the mechanism of the influence of specific bacteria and their metabolites. Because the gut microbiota has a sensitive dynamic response to diet and exercise, controlling the changes in these factors is the focus of future research.

## Figures and Tables

**Table 1:** Characteristics and differences of different dietary structures.

Dietary Pattern	Core Principles	Main Foods	Characteristics	Reference
MD	Based on traditional eating patterns of Mediterranean regions, emphasizing whole foods, healthy fats, and social dining.	High intake: Olive oil (primary fat source), whole grains, fruits, vegetables, legumes, nuts, and spices. Moderate intake: Fish (rich in Omega-3), dairy (yogurt, cheese), eggs, and optional red wine. Limited: Red meat, processed foods, and added sugars. Restrictions: Saturated fats (e.g., butter), refined sugars, and processed meats.	Focus on lifestyle (e.g., mindful eating, social meals) with olive oil as a staple.	[88,89]
MIND diet	Combines Mediterranean and DASH diets, specifically targeting brain health and preventing cognitive decline.	Emphasized: Leafy greens (≥6 servings/week), berries (≥2 servings/week), nuts (≥5 servings/week), whole grains, fish (≥1 serving/week), olive oil, and legumes. Limited: Butter (<1 tbsp/day), cheese (<1 serving/week), red meat (<4 servings/week), fried foods (<1 serving/week), and sweets.	Prioritizes brain-boosting foods (berries, greens) and strictly avoids trans fats and processed foods.	[90]
DASH diet	Reduces blood pressure through sodium control and balanced nutrition.	High intake: Vegetables, fruits, whole grains, and low-fat dairy. Moderate intake: Lean proteins (poultry, fish), legumes, and nuts. Limited: Salt (≤2300 mg sodium/day, ideally ≤1500 mg), red meat, sugary drinks, and saturated fats (e.g., full-fat dairy).	Strict sodium limits, with emphasis on potassium (bananas, potatoes), calcium (low-fat dairy), and magnesium-rich foods.	[91]
Plant-based diet	Primarily plant-derived foods, with minimal or no animal products (e.g., vegan).	Foundation: Vegetables, fruits, whole grains, legumes, nuts, seeds, and plant oils (e.g., coconut oil, olive oil). Optional: Dairy and eggs (for lacto–ovo vegetarians). Avoided: Strict versions exclude all animal products (meat, dairy, eggs, honey).	Highly flexible but requires attention to nutrients (e.g., vitamin B12, iron, Omega-3).	[92,93]

MD, Mediterranean diet; DASH, dietary approaches to stop hypertension; MIND, Mediterranean dietary approaches to stop hypertension intervention for neurodegenerative delay.

## Data Availability

The original contributions presented in the study are included in this article; further inquiries can be directed to the corresponding author.
